# Suppressing Systemic Interference in fNIRS Monitoring of the Hemodynamic Cortical Response to Motor Execution and Imagery

**DOI:** 10.3389/fnhum.2018.00085

**Published:** 2018-03-05

**Authors:** Shijing Wu, Jun Li, Lantian Gao, Changshui Chen, Sailing He

**Affiliations:** ^1^School of Information and Optoelectronic Science and Engineering, South China Normal University (SCNU), Guangzhou, China; ^2^Guangdong Provincial Key Laboratory of Optical Information Materials and Technology, Center for Optical and Electromagnetic Research, South China Academy of Advanced Optoelectronics, South China Normal University (SCNU), Guangzhou, China

**Keywords:** fNIRS, motor execution, motor imagery, systemic physiological interference, data preprocessing algorithm

## Abstract

Hemodynamic response to motor execution (ME) and motor imagery (MI) was investigated using functional near-infrared spectroscopy (fNIRS). We used a 31 channel fNIRS system which allows non-invasive monitoring of cerebral oxygenation changes induced by cortical activation. Sixteen healthy subjects (mean-age 24.5 yeas) were recruited and the changes in concentration of hemoglobin were examined during right and left hand finger tapping tasks and kinesthetic MI. To suppress the systemic physiological interference, we developed a preprocessing procedure which prevents over-activated reporting in NIRS-SPM. In the condition of ME, more activation was observed in the anterior part of the motor cortex including the pre-motor and supplementary motor area (pre-motor and SMA), primary motor cortex (M1) and somatosensory motor cortex (SMC; *t*_(15)_ > 2.27), however, in the condition of MI, more activation was found in the posterior part of motor cortex including SMC (*t*_(15)_ > 1.81), which is in line with previous observations with functional magnetic resonance imaging (fMRI).

## Introduction

Functional near-infrared spectroscopy (fNIRS; Jöbsis, 1977; Lloyd-Fox et al., 2010) is a non-invasive optical technique that uses either continuous, intensity-modulated, or pulsed near infrared light to monitor oxyhemoglobin (HbO_2_), deoxyhemoglobin (HbR) and total hemoglobin (HbT) in the cerebral cortex. With low-cost, safety, high temporal resolution and acceptable spatial resolution, fNIRS has been widely adopted to record brain activation in response to motor execution (ME) and motor imagery (MI) with potential applications in more naturalistic social environments than other brain computer interfaces (BCI; Coyle et al., 2007; Sitaram et al., 2007; Doud et al., 2011; Naseer and Hong, 2013; Koo et al., 2015; Acqualagna et al., 2016). Compared with EEG-based BCI, fNIRS-based is more sensitive to the localized activation, but slower due to the nature of metabolic response (Coyle et al., 2007; Sitaram et al., 2007). In the most general sense, MI refers to the “mental rehearsal of a simple or complex motor act that is not accompanied by overt body movements” (Solodkin et al., 2004). MI corresponds to a motor preparation process where motor programs are recruited to simulate motor performance without executing the movement. This so-called “simulation hypothesis” has been well established by psychophysiological (Decety et al., 1989; Pfurtscheller et al., 1997; Danckert et al., 2002) and neuroimaging studies in human subjects (Lotze et al., 1999; Naito et al., 2002; Ehrsson et al., 2003; Solodkin et al., 2004).

A number of studies using the functional magnetic resonance imaging (fMRI) or positron emission tomography (PET) have observed that MI activates several cortical regions similar to those activated by ME (Boecker et al., 2002; Lacourse et al., 2005; Hanakawa et al., 2008; Guillot et al., 2009). The cortical areas of ME and MI involved include the contralateral premotor area, primary motor cortex (M1), premotor cortex (PMC), supplementary motor area (SMA), anterior cingulate cortex (ACC), inferior and superior parietal lobule (IPL/SPL) and the cerebellum (CB; Grezes and Decety, 2001; Hétu et al., 2013).

Recently, fNIRS has been used for MI study and its further application on BCI and neurofeedback. However, the fNIRS results achieved so far regarding hemodynamic signal changes induced by MI are not very consistent. For example, in a study (Wriessnegger et al., 2008) higher oxygenation was observed in the motor areas (M1) as compared to the prefrontal cortex (PFC) for ME, but not for MI. Whereas, An et al found that MI induced a moderate activation in the M1. They also observed differences between ME and MI in the activation of the bilateral and lateral regions (An et al., 2013). Some studies have reported that cortical hemodynamic changes are more pronounced over the contralateral cortex (Watanabe et al., 1996; Hirth et al., 1997; Horovitz and Gore, 2003; Strangman et al., 2003; Holper et al., 2009; Leff et al., 2011). However, typical activation patterns have also been found over the ipsilateral cortex, Wriessnegger et al. (2008) found the oxygenation level was bilaterally represented for both tasks but with temporal differences.

These inconsistencies need to be addressed for efficient fNIRS-based BCI classification and feedback training. A possible reason for the inconsistency in fNIRS measurements might be due to the systematic physiological drift on the measured optical signals for a variety of reasons, including cardiac pulsations (~1–2 Hz), respiration (~0.2–0.4 Hz), Mayer waves (~0.1 Hz) and other very low-frequency fluctuations (0.01–0.05 Hz; Boas et al., 2004; Zhang et al., 2015). The amplitude of the systematic physiological drift is often comparable to that of the signal associated with brain activation (Gagnon et al., 2011). A band-pass filter can remove some components of the systemic interference such as those arising from cardiac pulsations and respiration. However, the low frequency components with frequency less than 0.1 Hz are usually mixed with the response hemodynamic signal, thus it cannot be eliminated by the filter, but deteriorating the response signal. Therefore, in fNIRS data analysis, it is critical to efficiently extract task-related hemodynamic signal in cortex from measured data mixed with the systemic interference.

The purpose of this study was to measure the hemodynamic response with fNIRS to motor tasks including ME and imagery. We expected by developing a suitable data analysis method, it might be possible to effectively suppress the systemic hemodynamic interference, thus uncovering the unbiased activation patterns induced by ME and MI, which would be similar to those revealed by fMRI studies. The result achieved will guide future motor-related fNIRS-based BCI.

## Materials and Methods

### Participants

Sixteen healthy participants (nine males and seven females) were recruited for this study. Participants ranged in age from 19 years to 52 years old (24.5 ± 7.4 years). All participants were right-handed according to a modified Edinburgh Handedness Questionnaire (Oldfield, 1971), and had no history of neurological or psychiatric disease. Written informed consents were obtained from all participants, and the protocol of the present study was approved by the Institutional Review Board of South China Normal University (No. 177).

### Experimental Procedure

**Figure 1:**
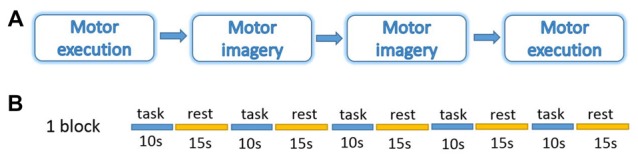
Experiment design. **(A)** Four blocks in a session. **(B)** Five trails of 10 s task and 15 s rest in each block. The experiment was performed under four conditions: motor execution (ME); motor imagery (MI) with the right or left hand.

During measurements, all the participants were asked to sit on a comfortable chair, place their hands on the table in front of them, and relax for about 5 min before the experiment to get rid of any existing hemodynamic response induced by their previous activation. The participants were instructed to perform motor tasks with four conditions in two different sessions: ME and MI with the right and left hand. The orders of the two sessions were counter-balanced (Figure 1). The experiment protocol was a block design including 20 trails of 10 s of task and 15 s of rest in each condition. During ME task, participants were instructed to tap the indicated hand once per second. During MI task, they were instructed to imagine the same actions as they performed during ME but refrain from any movement. They were also instructed to use kinesthetic imagery that requires individuals to “feel the movement”, i.e., to perceive the sensations associated with its execution such as muscle stretching and contractions (Hétu et al., 2013). During the rest period, participants were instructed to relax, refrain from moving, and not think about anything in particular. Prior to the measurements, subjects practiced both ME and MI tasks, guided by the experimental program and experimenter, in order to familiarize themselves with the protocol and tasks.

### fNIRS Measurements

**Figure 2:**
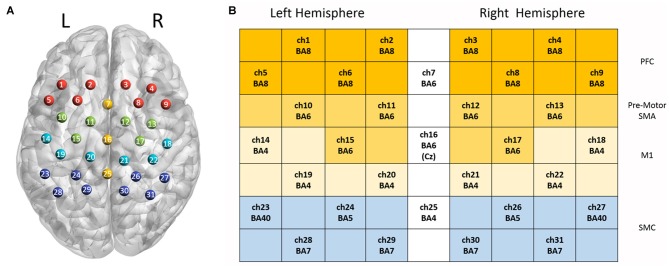
Channel configuration of the optical probes. Panel **(A)** illustrates the locations of 31 optical channels. Panel **(B)** illustrates the measured anatomical regions and the corresponding Brodmann areas (BAs).

In this study, 31 channels of a commercial continuous-wave (CW) fNIRS image system (FOIRE-3000, Shimadzu Corporation, Kyoto, Japan) were used to measure the cortical activity of the parietal lobe. The absorption of three wavelengths (780 nm, 805 nm and 830 nm) of near infrared light were measured with a sampling rate of 7.14 Hz and then transformed into concentration changes of the HbO_2_, HbR and HbT by the modified Beer-Lambert law (Owenreece et al., 1999). The optical probes consisted of 10 sources and 10 detectors, building up 31 channels. We determined the probe locations by the international 10-10 system (Koessler et al., 2009), which were further confirmed by a 3D digitizer (FASTRAK-Polhemus, Polhemus, VT, USA) and NIRS-SPM (Ye et al., 2009). We applied the 3D digitizer to record the exact spatial coordinates of four reference points of the 10-10 system (NZ, CZ, AL, RL), as well as the 20 optical probes. We converted these coordinates into locations of the 31 channels in an estimated MNI space by NIRS-SPM (Tsuzuki et al., 2007; Ye et al., 2009). With these probe locations in MNI, we used BrainNet Viewer (Xia et al., 2013) to generate Figure 2 in which all optical channels were shown on the measured brain regions. Since NIRS-SPM gave a set of probability values for the measured anatomical region of each channel, we chose 80% probability as the threshold (Zhu et al., 2017) to determine the channel location. The area of arrays of optodes was 9 cm × 12 cm with the inter-optode distance of 3 cm.

### Data Preprocessing and Statistical Analysis

#### Preprocessing

**Figure 3:**
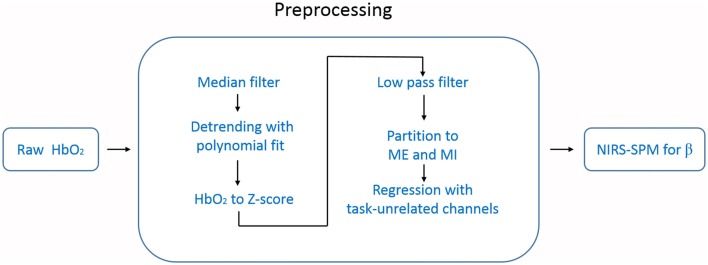
Flow chart of the data processing. The data preprocessing includes six steps before NIRS-SPM.

Directly using the NIRS-SPM to analyze the raw temporal data (e.g., HbO_2_) resulted in over-activation. We thought this came from the systemic interference whose time scale was overlapped with our task period (~25 s). To suppress this systematic drift, we designed a preprocessing procedure before using NIRS-SPM. The data processing steps are schematically illustrated in Figure 3. For each measurement channel, we applied a one-dimensional median filter (window width = 3) to the raw temporal data of HbO_2_ to eliminate outliers such as those with sudden jumps or drops. Then the temporal data were detrended with a second order polynomial fit to remove the slow drift. Each time-series of HbO_2_ was converted to its Z score (i.e., Z = (HbO_2_ − mean (HbO_2_))/std (HbO_2_)), a measure of data with its own variance. This conversion is important for achieving an unbiased group average for the hemoglobin data recorded by our fNIRS setup (FOIRE 3000). Because the data provided by the FOIRE 3000 includes an unknown parameter L as a multiplying factor (e.g., LHbO_2_). L is the photon average path length from the source to the detector, which does not only depend on the wavelength, but also varies from subject to subject, even from channel to channel for the same subject. By normalizing with its own variance, the unknown parameter L is canceled out in the Z score.

To get rid of task-unrelated systemic components, such as cardiac cycles (~1 Hz), venous pressure waves due to respiration (~0.2 Hz) and arterial pressure oscillations (Mayer waves ~0.1 Hz), we applied a low pass filter (zero-phase second order Butterworth) with the cutoff frequency of 0.08 Hz to the time-series of Z score (White et al., 2009; Mesquita et al., 2010).

After the steps above, we divided data into two groups: ME and MI according to each type of marks made during the measurement. In our experimental design, each trail consisted of 10 s task followed by 15 s rest period. Thus, the expected response frequency is around 0.04 Hz, which is in the low frequency band (e.g., <0.1 Hz) in which there are plenty of undetermined hemodynamic components. To suppress the potential adverse influence of these components on the task-related response, we designed a regression method in which the task-unrelated systemic hemodynamic components were removed. In this regression method, we calculated the temporal correlation function between each channel and the task function (a squared-waveform is equal to 1 when task is on, 0 when the task is off), and then selected task-unrelated channels identified by low correlation with the task (absolute value of the correlation coefficient <0.2). The average value of these task-unrelated channels was taken as the regressor. We performed this regression procedure for each hemisphere separately, the left regressor was estimated from channels located in the left hemisphere, and used only for the left hemisphere, and vice versa.

**Figure 4:**
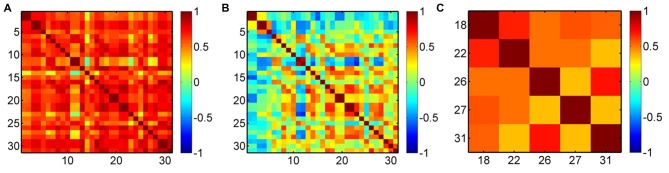
Correlation maps of oxyhemoglobin (HbO_2_) for a subject performing the left hand execution task. **(A)** Before the preprocessing, **(B)** after the preprocessing and **(C)** correlation map for the activation channels (Ch18, 22, 26, 27, 31) after the regression. The numbers along the *x*-axis and *y*-axis indicate the optical channels. Each pixel value is the correlation coefficient between the two corresponding channels.

To demonstrate the efficacy of the regression, we present an example in Figure 4. Before the regression, most of the measurement channels are highly correlated (Figure 4A), which was possibly due to the systemic interference mixed in each channel. Therefore, if the removal of the systemic drift is not very effective, such as our case where the time scale of the task period (~25 s) overlaps with the systemic hemodynamic oscillations, there must be an overall effect on the result of the cortical hemodynamic response. After the regression, the overall correlation was reduced (Figure 4B), but the activation channels (e.g., Ch18, 22, 26, 27 and 31 in this example for the left hand execution) locating at the right M1 and somatosensory motor cortex (SMC) were still correlated (Figure 4C). This implies that the regression did suppress the systemic drift, but had no (or less) influence on the activation channels.

After the regression, the processed data which had less (or no) influence from the systematic interference, were sent to the public software NIRS-SPM to get the β value for each block of tasks. The β value indicates the magnitude of activation during task period in each block. By averaging β values across 10 blocks for each task, we obtained mean activation magnitude for the ME and imagery. The t-value for each channel was obtained by performing the Student-*t*-test over all subjects. For visualizing the activation, a pseudo-colored t-map was plotted for each task to show the activation pattern.

Since the HbR signal has lower signal to noise ratio, in the data analysis, we only analyzed HbO_2_.

#### Statistical Analysis

To investigate the activation of four different conditions including ME and MI in both hands, we applied a one sample *t*-test to the β values with and without the preprocessing, including a multiple-testing correction based on Benjamini false discovery rate (FDR; Benjamini and Hochberg, 1995).

Two-way repeated ANOVA was employed to investigate the effect of task (ME vs. MI) and hand (left hand vs. right hand) in each regions of interest (ROIs), and then a *post hoc* test was performed with paired *t*-test (with Bonferroni correction). The ROIs were defined as follows: Ch1, 2, 5, 6 (left hemisphere) and Ch3, 4, 7, 8 (right hemisphere) corresponding to prefrontal regions (PFC); Ch10, 11, 15 (left hemisphere) and Ch12, 13, 17 (right hemisphere) correspond to the pre-motor and supplementary motor area (pre-motor and SMA); Ch14, 19, 20 (left hemisphere) and Ch18, 21, 22 (right hemisphere) correspond to the M1; Ch23, 24, 28, 29 (left hemisphere) and Ch26, 27, 30, 31 (right hemisphere) correspond to the SMC. The Statistical Product and Service Solutions (SPSS) software package was used for statistical analysis, and statistical significance level was defined as *p* < 0.05.

## Results

### Activation in the Four Conditions

**Figure 5:**
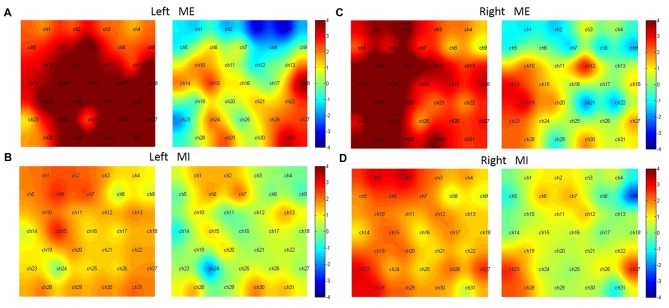
Hemodynamic activation maps with and without the preprocessing. Left ME **(A)**, Left MI **(B)**, right ME **(C)** and right MI **(D)**. For comparison, in each sub-figure the left panel shows activation without the preprocessing, the right shows activation with the preprocessing.

In the condition of Left ME (Figure 5A), without the preprocessing, almost all channels were activated except Ch1, 4, 23; while with the preprocessing, the activated channels included Ch15, 18, 27, 29, 31 (*t*_(15)_ = 2.43, 3.56, 2.45, 2.27, 3.27, *p* < 0.05, FDR corrected), corresponding to the left pre-motor, SMA, M1, SMC and right M1, SMC.

In the condition of Left MI (Figure 5B), without the preprocessing, the activated channels were Ch2, 6, 7, 15; However, with the preprocessing, no channels were statistically active. Two channels (Ch7 and 30), corresponding to the center of pre-motor and right SMC, were marginally activated (*t*_(15)_ = 1.90, 1.81, *p* < 0.1).

In the condition of Right ME (Figure 5C), without the preprocessing, almost all channels were activated except Ch4, 8, 9, 22 and 25; With the preprocessing, Ch12, 14, 19, 23 were activated (*t*_(15)_ = 2.70, 3.00, 3.13, 2.43, *p* < 0.05, FDR corrected), corresponding to the right M1, left pre-motor and SMA and SMC.

In the condition of Right MI (Figure 5D), without the preprocessing, Ch1, 2, 6, 7, 15, 19, 23, 27, 28 were activated; with the preprocessing, Ch23, 27 were activated (*t*_(15)_ = 2.58, 2.54, *p* < 0.05, FDR adjusted), corresponding to the bilateral SMC.

The results showed that in the condition of ME, more activation was observed in the anterior part of the motor cortex including the pre-motor, SMA, M1 and SMC, however, in the condition of MI, more activation was observed in the posterior part of the motor cortex, namely, the SMC.

### Overlap

For the conditions of the left hand task, although there were no overlapping channels activated significantly by both ME and MI, Ch31 activated by ME, and Ch30, activated by MI are both located in the SMC. Therefore, for the left hand task, the right SMC was an overlapping area activated by both ME and MI. For the right hand task, the only overlapping channel activated by both ME and MI was Ch23, locating at the left SMC.

### Lateralization

A significant main effect of the task was found in the left PFC and right PFC (left PFC: *F*_(1,15)_ = 7.02, *p* = 0.02, $\eta_{\text{p}}^{2}$ = 0.32; right PFC: *F*_(1,15)_ = 8.63, *p* = 0.01, $\eta_{\text{p}}^{2}$ = 0.37). The *post hoc* test indicated that the HbO_2_ levels were more active during the task of MI compared to the task of ME in the PFC (*mean difference* (MD) = 0.04, *p* = 0.02; MD = 0.04, *p* = 0.01, respectively); whereas no main effects of the hand were observed in the PFC (left PFC: *F*_(1,15)_ = 1.16, *p* = 0.30, $\eta_{\text{p}}^{2}$ = 0.07; right PFC: *F*_(1,15)_ = 1.52, *p* = 0.24, $\eta_{\text{p}}^{2}$ = 0.09), and no interaction effects were significant for the task and hand in the PFC (left PFC: *F*_(1,15)_ = 0.00, *p* = 0.96, $\eta_{\text{p}}^{2}$ < 0.00; right PFC: *F*_(1,15)_ = 2.14, *p* = 0.16, $\eta_{\text{p}}^{2}$ = 0.13).

In the pre-motor and SMA, only one significant main effect was observed in the left SMA (left SMA: *F*_(1,15)_ = 5.31, *p* = 0.04, $\eta_{\text{p}}^{2}$ = 0.26; right SMA: *F*_(1,15)_ = 0.09, *p* = 0.77, $\eta_{\text{p}}^{2}$ = 0.01). The *post hoc* test indicated that in the left SMA, the HbO_2_ level was more active during the ME compared to MI (MD = 0.03, *p* = 0.04); no main effects of the hand were observed in the pre-motor and SMA (left SMA: *F*_(1,15)_ = 0.10, *p* = 0.76, $\eta_{\text{p}}^{2}$ = 0.01; right SMA: *F*_(1,15)_ = 0.37, *p* = 0.55, $\eta_{\text{p}}^{2}$ = 0.02), and no interaction effects were significant for the task and hand in the SMA (left SMA: *F*_(1,15)_ = 0.30, *p* = 0.59, $\eta_{\text{p}}^{2}$ = 0.02; right PFC: *F*_(1,15)_ = 0.60, *p* = 0.45, $\eta_{\text{p}}^{2}$ = 0.04).

**Figure 6:**
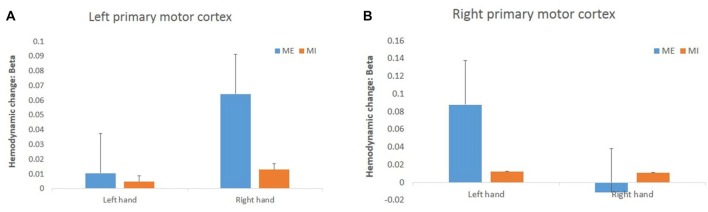
Changes in HbO_2_ concentration (in terms of β value) during the task in the left **(A)** and right **(B)** primary motor cortex (M1). The HbO_2_ level of the primary motor cortex increased significantly only during the right ME but not during MI.

For the hemodynamic changes in the M1 (Figure 6), the HbO_2_ level in the right M1 increased significantly during the contralateral ME, and there was a significant interaction effect between task and hand (*F*_(1,15)_ = 11.34, *p* = 0.00, $\eta_{\text{p}}^{2}$ = 0.43). In the left M1 there was no interaction effect between task and hand (*F*_(1,15)_ = 1.74, *p* = 0.21, $\eta_{\text{p}}^{2}$ = 0.10). There were no main effects of hand and task observed in left M1 (task: *F*_(1,15)_ = 2.91, *p* = 0.11, $\eta_{\text{p}}^{2}$ = 0.16, hand: *F*_(1,15)_ = 2.95, *p* = 0.11, $\eta_{\text{p}}^{2}$ = 0.17) and right M1 (task: *F*_(1,15)_ = 1.95, *p* = 0.18, $\eta_{\text{p}}^{2}$ = 0.12, hand: *F*_(1,15)_ = 2.89, *p* = 0.11, $\eta_{\text{p}}^{2}$ = 0.16).

In addition, neither main effect nor interactions were significant for the left SMC (task: *F*_(1,15)_ = 0.53, *p* = 0.48, $\eta_{\text{p}}^{2}$ = 0.03, hand: *F*_(1,15)_ = 1.42, *p* = 0.25, $\eta_{\text{p}}^{2}$ = 0.09; interaction: *F*_(1,15)_ = 1.84, *p* = 0.20, $\eta_{\text{p}}^{2}$ = 0.11) and right SMC (task: *F*_(1,15)_ = 1.44, *p* = 0.26, $\eta_{\text{p}}^{2}$ = 0.09, hand: *F*_(1,15)_ = 0.95, *p* = 0.35, $\eta_{\text{p}}^{2}$ = 0.06; interaction: *F*_(1,15)_ = 0.59, *p* = 0.45, $\eta_{\text{p}}^{2}$ = 0.04).

## Discussion

In the present study, we measured hemodynamic changes with fNIRS during ME and MI of a simple finger tapping task. In the condition of ME, the activation areas included the pre-motor and SMA, M1 and SMC. In the condition of MI, the SMC and posterior of the motor cortex were activated. This observation has not been reported in fNIRS, but is consistent with fMRI studies.

### Data Preprocessing

In the present study, we adopted an experimental protocol with 10 s as the task and 15 s as the rest period. For data analysis, we first used the publicly available software NIRS-SPM (Tak et al., 2011) to analyze the raw fNIRS data. NIRS-SPM is based on the general linear model (GLM), widely used for analyzing time-series of fNIRS data. In this software, to get rid of possible systemic interference, a detrending algorithm named wavelet-MDL (minimum description length) was used. The β value of the GLM for different trials were extracted and averaged to account for the brain activity. However, we found that directly using NIRS-SPM to analyze our data resulted in over-activation (see Figure 5) due to the interference of the systemic hemodynamic component. Removing systemic hemodynamics is difficult since its time scale overlaps with the task period. This interference also obscured the real activation patterns, such as that in MI (Figure 5D). Though the wavelet-MDL detrending algorithm was developed and has been demonstrated to be useful in eliminating the systemic interferences (Tak et al., 2011), we found it was not very effective in processing our data (see left column of each subfigure in Figure 5). It is probably because that the central frequency of our response signal is too close to that of the low frequency components of the systemic interference. By preprocessing the data with our preprocessing method prior to using NIRS-SPM, the activation was revealed, which was consistent with the fMRI observation. Considering our data with and without preprocession, it is plausible that the inconsistency in fNIRS studies on ME and MI might come from the interference of the systemic hemodynamic oscillation, which is hard to remove, and needs to be treated very carefully.

### Motor Imagery

Previous studies have shown that the cortical response to MI depends on the type of MI. There are two primary types of the MI: visual imagery and kinesthetic imagery. In the scenario of visual imagery, the subject self-visualizes the movement; while in the kinesthetic imagery, the subject imagines the feelings and sensations produced by the movement (Batula et al., 2017). A meta-analysis suggested that kinesthetic tasks may increase more activation in motor and associated areas (Hétu et al., 2013). Therefore, in the present study we chose the kinesthetic imagery as imagery task, and observed the activation in the SMC and posterior of the motor cortex.

### PFC

For the four conditions, there was less activation in terms of HbO_2_ in PFC as compared to the other ROIs; however, we observed more activation in the condition of MI compared to the ME (Figure 5). This indicates that the PFC plays a more important role in MI than ME, especially the left PFC. This observation is in line with previous studies (Fiehler et al., 2007; Vry et al., 2012) reporting that imagery-specific cognitive functions are implemented in the ventral system. Since the MI is a subliminal cognitive process, it is associated with short-term maintenance of kinesthetic information. The kinesthetic working memory involves a neural network that activates the ventrodorsal part of the left hemisphere (Fiehler et al., 2007). The networks include the dorsolateral prefrontal cortex (DLPFC), which plays an important role in keeping MI as the representations of a given motor act are internally prepared during imagery.

### Pre-Motor and SMA

We observed that the pre-motor and SMA were activated only in the ME. This result is similar to those observed in previous studies, in which the activation was measured by PET or fMRI (Stephan et al., 1995; Hikosaka et al., 1996; Ruby and Decety, 2003; Solodkin et al., 2004).

SMA is known to be involved in ME. Studies in primates have shown that the ventral and dorsal premotor cortices play important roles in the planning, preparation and execution of motor acts (Hoshi and Tanji, 2007; Hétu et al., 2013). Previous imaging studies have shown that the activity in SMA is directly related to movement output (Obrig et al., 1996; Christensen et al., 2000). It has been reported that the SMA is involved in motor preparation and is activated not only during ME, but also during the preparation and inhibition of movements (Kasess et al., 2008; Guillot et al., 2012; Iso et al., 2016). Some studies also suggest that MI requires a similar amount of time as the execution does (Guillot and Collet, 2005), implying that they are produced through the analogous computational steps in the brain (Hétu et al., 2013). However, some studies suggest that the SMA is responsible not only for the preparation and execution of intended movements, but also for suppressing movements that are represented in the motor system, but not to be performed (Enzinger et al., 2008; Kasess et al., 2008). This may explain why the SMA is only activated in ME but not in MI.

### M1

In the present study, the M1 was activated in the contralateral hemisphere during ME, but not activated during MI.

In the condition of ME, even though there is only a significant interaction effect in right M1, we can see from Figure 5A that the right hand ME induces a more significant hemodynamic change than the left hand ME. Whether the M1 is consistently activated during MI is a continuing argument. In some studies, the activation was found in the M1 during MI, but in other studies it was not. In a meta-analysis review, Hétu et al. (2013) have noted that, though the MI seems to use similar structures as ME, M1 is not consistently activated during MI.

Numerous transcranial magnetic stimulation (TMS) studies have provided strong evidence that MI can enhance the excitability of the M1 (Menz et al., 2009; Loporto et al., 2011), which implies that there might be a link between MI and function of the M1. However, some fMRI and PET studies have indicated that there is no activation in the M1 during MI (Binkofski et al., 2000; Gerardin et al., 2000; Hanakawa et al., 2002). This is consistent with what we observed. The reason that MI is not activated during MI might be due to the fact that the SMA exerts an inhibitory influence on the M1 during kinesthetic MI (Kasess et al., 2008). On the other hand, there is individual difference in response to MI. Some studies have reported that although there is no group activation in the M1, single-subject analysis may clearly show activation in the M1 in a few participants (Gerardin et al., 2000; Dechent et al., 2004; Hétu et al., 2013), which in fact was observed in the present study. Individual characteristics that affect the cortical response to the MI may include motor expertise (Milton et al., 2008; Chang et al., 2010), age (Skoura et al., 2009; Personnier et al., 2010), sex (Schuster et al., 2011), and experience/practice (Guillot et al., 2008; Malouin and Richards, 2010; Hétu et al., 2013). Various factors may exert influence on the activation of M1 during MI, which needs further exploration and clarification.

### SMC

In this study, we found that ME had more activation in the anterior part of the motor cortex. However, in the condition of MI, more activation was observed in the posterior part of motor cortex. Moreover, for the left hand task, the right SMC was an overlapping area activated by both ME and MI, while for the right hand task, the overlapping channel activated by both ME and MI was Ch23, locating at the left SMC.

Numerous imaging studies have observed that there is an activation in the SPLs (BA 7) or inferior parietal lobules (BA 40) in participants performing MI tasks (Decety et al., 1994; Stephan and Frackowiak, 1996), which supports the results we observed. The specific role of the SMC in MI that emerges here is consistent with neuropsychological studies. For example, Sirigu et al. (1996) demonstrated that patients with parietal lesions lose the ability to predict duration of a movement through mental rehearsal, contrary to normal subjects and patients with damage in the primary motor area. Moreover, patients with left parietal lesions were impaired when imagining movements of both the left and the right hand, while patients with right parietal lesions only showed an imagination deficit of the contralateral hand (Gerardin et al., 2000). Thus, the SMC could play an important role in MI.

During the MI, though we carefully observed the hand of subjects to ensure that there is no movement-related data for the analysis of MI, however, due to the lack of Electromyographic (EMG) recordings on the muscle, slight and invisible movement of fingers might be ignored. EMG is a useful tool for monitoring and rating muscle movement, however, fMRI studies have demonstrated EMG signal is not well correlated with the activation of the associated motor cortex (Porro et al., 1996; Wriessnegger et al., 2008). Therefore it is not very likely that EMG is appropriate measure for grading the MI which is eventually reflected by the cortical hemodynamics. In our fNIRS recording, the signal-to-noise ratio was poor for HbR, thus we did not analyze HbR for each task. This might be another limitation of this work.

## Conclusion

In summary, we used fNIRS to measure the cortical response to ME and MI. To analyze the measured fNIRS data, we used a task-unrelated regression algorithm prior to the NIRS-SPM to suppress the systemic interference. With this data analysis method, we observed that ME induced more activation in the anterior portion of the motor cortex, including the pre-motor and supplementary motor, primary motor and somatosensory cortices. MI induced more activation in the posterior part of the motor cortex, such as the somatosensory cortex. These observations were in line with previous fMRI studies.

## Author Contributions

SW, JL, CC and SH designed the study. SW and LG performed the experiments. SW, JL and SH analyzed the data and wrote the article.

## Conflict of Interest Statement

The authors declare that the research was conducted in the absence of any commercial or financial relationships that could be construed as a potential conflict of interest.
